# Application and efficacy of levetiracetam in prophylactic treatment of migraine without aura

**DOI:** 10.1186/1129-2377-1-S14-P207

**Published:** 2013-02-21

**Authors:** TN Maykova

**Affiliations:** 1 Hedache Center Kyiv, Ukraine

## Introduction

In connection with necessity of extension of therapeutic possibilities of migraine treatment the study of efficacy of Levetiracetam for profilactic treatment was conducted. There are not data in literature on undertaking big randomized studies on this topic.

## Background

With a glance for pharmacotherapeutic peculiarities of action of traditional anticonvulsants, that limit their application due to side-effects (valproic acid and topiramate are meant), there is a necessity of enlargement of the range of drugs, that can be used for the prophylactic treatment of migraine.

## Objectives and methods

The study was conducted on the base of Headache Center on patients that applied with migraine without aura [1] with attacks resistant to NSAID or triptans, or frequent attacks (4-8 per month). Patients were randomized on gender, age, duration of the disease. 30 patients took Levetiracetam (1000 mg/day) like prophylactic treatment for 6 months, 30 patients took valproic acid (1000 mg/day) for 6 months, and 30 patients took Topiramate (200 mg/day) for 6 months. Efficacy of the treatment was assessed by quantity of days with headache during the month, quality of life index [2].

## Results

Levetiracetam showed efficacy comparable with traditionally used anticonvulsants. The efficacy of Levetiracetam was unreliably lower than the efficacy of valproic acid, and it was reliably higher than the efficacy of Topiramate.
